# Discrimination of *Gastrodia elata* from Different Geographical Origin for Quality Evaluation Using Newly-Build Near Infrared Spectrum Coupled with Multivariate Analysis

**DOI:** 10.3390/molecules23051088

**Published:** 2018-05-04

**Authors:** Yamin Zuo, Xuehua Deng, Qing Wu

**Affiliations:** 1 Guizhou Key Laboratory for Information System of Mountainous Areas and Protection of Ecological Environment, Guizhou Normal University, 116 Baoshan North Rd, Guiyang 550001, Guizhou, China; 20170529@hbmu.edu.cn; 2 School of Basic Medical Sciences, Hubei University of Medicine, 30 Renmin South Rd, Shiyan 442000, Hubei, China; 3 School of Pharmacy, Hubei University of Medicine, 30 Renmin South Rd, Shiyan 442000, Hubei, China; dxhhbmu@163.com; 4 Innovation Laboratory, the Third Experiment Middle School in Guiyang, Guiyang 550001, Guizhou, China

**Keywords:** near infrared spectroscopy, *Gastrodia elata*, quality evaluation, phenolic compounds content, geographical origin, multivariate analysis

## Abstract

Discrimination of *Gastrodia elata* (*G. elata*) geographical origin is of great importance to pharmaceutical companies and consumers in China. this paper focuses on the feasibility of near infrared spectrum (NIRS) combined multivariate analysis as a rapid and non-destructive method to prove its fit for this purpose. Firstly, 16 batches of *G. elata* samples from four main-cultivation regions in China were quantified by traditional HPLC method. It showed that samples from different origins could not be efficiently differentiated by the contents of four phenolic compounds in this study. Secondly, the raw near infrared (NIR) spectra of those samples were acquired and two different pattern recognition techniques were used to classify the geographical origins. The results showed that with spectral transformation optimized, discriminant analysis (DA) provided 97% and 99% correct classification for the calibration and validation sets of samples from discriminating of four different main-cultivation regions, and provided 98% and 99% correct classifications for the calibration and validation sets of samples from eight different cities, respectively, which all performed better than the principal component analysis (PCA) method. Thirdly, as phenolic compounds content (PCC) is highly related with the quality of *G. elata*, synergy interval partial least squares (Si-PLS) was applied to build the PCC prediction model. The coefficient of determination for prediction (R_p_^2^) of the Si-PLS model was 0.9209, and root mean square error for prediction (RMSEP) was 0.338. The two regions (4800 cm^−1^–5200 cm^−1^, and 5600 cm^−1^–6000 cm^−1^) selected by Si-PLS corresponded to the absorptions of aromatic ring in the basic phenolic structure. It can be concluded that NIR spectroscopy combined with PCA, DA and Si-PLS would be a potential tool to provide a reference for the quality control of *G. elata.*

## 1. Introduction

*Gastrodia elata* (*G. elata*), is a medicine food homology product in China, whose roots were traditionally used as a traditional Chinese medicine (TCM) with treatment of headaches, neuralgia, dizziness, hypertension and other related neuralgic disorders [1,2,3]. Now it is also used as one sub-material in food and Chinese Patent Medicines (CPM) widely. As the wild *G. elata* is not sufficient enough for commercial large-scale exploitation, its artificial cultivation medicine has become essential to meet the increasing requirement of markers. The herb is mainly planted in the central and southwestern regions in China [4], such as Yichang (Hubei), Hanzhong (Shanxi), Dabieshan (Anhui) and Zhaotong (Yunnan). Many studies have indicated that the efficacy and quality of herbal medicines are somewhat different depending on cultivation soil and climate, based on geographic origin, even when coming from the same species [5,6]. 

Currently, the approaches for detecting the origin of *G. elata* are mainly sensory analysis and analytical methods. The former depends basically on the experience and personal emotion of *G. elata*, which is not objective and lacks scientific understanding; the latter is restricted to the detection of a few active components using methods such as HPLC(LC-MS), GC(GC-MS) and capillary electrophoresis [7,8,9], which are often labor-intensive, expensive, time-consuming and require large amounts of organic solvent. In the end, the quality value of the tested sample is determined as “qualified” or “unqualified” according to the presenting acceptance criteria [10]. Considering these potential quality risks and to ensure the safety and uniformity for pharmaceutical companies and consumers, there is a significant interest in establishing a rapid and effective method for authenticating the origin of *G. elata*.

Near infrared spectrum (NIRS) is widely used as alternatives to wet chemistry procedures for qualitative and quantitative analysis, which can be a rapid and effective instrument to classify different origin of *G. elata* coupled with chemometrics; since it is fast, easy to handle and non-destructive, it has been applied to classify coffee varieties, milk power and Chinese herbs [11,12,13]. However, few studies explore the identification of the cultivation origins of *G. elata*. Some studies have proved that NIR spectroscopy coupled with supervised pattern recognition can be applied to provide qualitative assessment, while to choose the most appropriate method still remains our consideration. In this study, we applied principal component analysis (PCA) to discuss the possibility of *G. elata* samples discrimination with NIR spectroscopy and other pattern recognition as discriminant analysis (DA) was applied and compared.

Furthermore, phytochemical studies of *G. elata* have validated that the presence of phenolic compounds in *G. elata*, for example, *p*-hydroxybenzyl alcohol (HA), gastrodin (GA) and parishins compounds have been given rise to more attentions because of their pharmacological features [14,15], it is inappropriate to choose only several specific components as essential criteria. Although many efforts have been endeavored to the quality evaluation of *G. elata* [16,17,18], further exploration is still required. Numerous studies have revealed that the phenolic compounds content (PCC) in *G. elata* is an important quality index when evaluating its medicinal efficacy [15,17,19]. For this application, the correlation between the phenolic compounds content in *G. elata* and NIRS was also developed, we explore synergy interval partial least squares (Si-PLS) to provide predictive models of PCC by selecting different subintervals.

Therefore, the objective of this study was to build a new method for quality evaluation of *G. elata* based on NIRS coupled with multivariate analysis, which concludes: (1) the traditional HPLC method was developed to simultaneously determine four phenolic compounds content in *G. elata* samples, which provided basic evaluation of their geographical origin and the numerical value in order to build the NIRS model; (2) to develop the classification models using PCA and DA for qualitative analysis, and compare the performance of two methods; and (3) the Si-PLS algorithm was used to choose the most efficient sub-interval spectrums for quantitative analysis of PCC in *G. elata*; in the end, the evaluation between HPLC and NIRS method were simply compared.

## 2. Experimental

### 2.1. Material and Reagents

All *G. elata* in our lab were supplied by local farmers or manufactures from four main-cultivated regions of China, namely: (1) the Yangtse Gorges and shennongjia area, (2) the Southwest provinces area, (3) the Dabieshan area, and (4) the Northeast area. Since the chemical components are generally similar among the samples from the same province [20], samples were acquired from two different provinces in each area during the 2017 and 2018 harvest seasons as shown in Table 1. So a total of 16 batches of *G. elata* were collected in this study and authenticated by Professor Wei Shenhua from Guiyang College of Traditional Chinese Medicine.

Reference standards (gastrodin, *p*-hydroxybenzyl alcohol, parishin A and parishin B) were supplied by China Food and Drug Testing Institute with the purity (≥98%) determined by HPLC-MS. Methanol and acetonitrile were purchased from Kemmo chemical reagent Co., Ltd. (Tianjin, China). Purified water comes from a Milli-Q water purification device (Millipore, Burlington, MA, USA). Other chemical reagents were all analytical grade.

### 2.2. Sample Preparation and Standard Solution for HPLC

Upon arrival, after washed cleanly the soil dust from *G. eleta* surface, the samples were put into an electric steamer (Midea Group, Foshan, China) immediately and heated about 15 min to imitate the steaming process used by manufactures, then spread out and dried in a force-draught oven (Dongguan Ke-chang Machine Co., Dongguan, Guangdong, China) at 50 °C for about 10 h. At last, the samples were ground into fine pieces and screened through a 20 mm sieve that were used for further analysis. All procedures in this study were strictly controlled to lower the risk of uncertain parameters during sample collection, transportation and pre-treatment.

To prepare the further experiment, about 0.5 g raw *G. elata* powder sample was weighed to be extracted with 25 mL of 50% alcohol then be ultrasounded for 30 min. Weighed again and the lost 50% alcohol was added then the solution was filtered on qualitative filter paper twice. The filtrate was collected and pipetted 10 mL of the subsequent filtrate to concentrated to dry, using the mixed solution of acetonitrile-water (3:97) to resolve residue and fixed the volume to a 10 mL volumetric flask. The extraction solution was filtered with 0.22 μm organic phase filtration membrane before traditional HPLC analysis. The chromatographic analysis was carried on an Agilent 1260 HPLC System comprising a diode-array detector.

The standard stock solutions used here were prepared in advance by dissolving the four reference standards in 50% methanol to a final concentration of 0.0591 mg/mL for gastrodin, 0.0055 mg/mL for *p*-hydroxybenzyl alcohol, 0.0315 mg/mL for parishin A, 0.0025 mg/mL for parishin B.

### 2.3. Sample Preparation and Measurement for NIRS

For each *G. elata* powder sample, about 0.5 g was weighed and densely packed into the sample cup then compressed it. The samples’ particle size was controlled below 0.25 mm. In this study, infrared spectra was collected on an Antaris II FT-NIR spectrophotometer (Thermo Scientific Co., Waltham, MA, USA) with an InGaAs detector (Thermo Scientific Co., USA). The sample container is designed specifically by Thermo Scientific Co. to hold samples. The NIRS measurements were performed within the region from 4000–10,000 cm^−1^, and the automatic sampler with the reflection mode was used to gain the spectroscopy automatically. Each sample spectrum was collected three times by an average of 64 scans with a resolution of 8 cm^−1^, all spectra were recorded as log(1/R), where R was the relative reflectance, the mean of three spectra was used in the following analyze step. To lower the risk of uncertain parameter and ensure a representative data, the temperature was controlled at 25 °C and the humidity remained at a steady level in our laboratory.

Before establishing the models, the spectral data should be transformed for an optimal performance. The data preprocessing methods on pattern recognition had been studied including Savitzky-Golay (SG) smoothing, multiplicative scatter correction (MSC), first and second derivative (F/SD) of raw spectrum, and standard normal variate (SNV) which can be used to optimize the model. For example, the SG method was applied to decrease noise, MSC and SNV can be used to correct the light scatter and reduce the path length change of light.

### 2.4. Chemometric for Geographical Origin Determination of G. elata

The aim of our study is to identify the geographical origin of *G. elata* by newly-built NIR spectrum method with suitable pattern recognition model. In this study, PCA and DA were explored in the classification of geographical origin, and Si-PLS method was used for prediction of PCC in *G. elata*.

#### 2.4.1. PCA

Principal Component Analysis (PCA) is a well-known method for variable reduction and transforming the original independent variables into new axes [21]. By plotting the PCAs, one can detect the possible spectral outliers and discriminate the samples based on the geographical origins. The first principal component (PC1) accounts for the maximum of the total variance, the second (PC2) is uncorrelated with the first and represented to the maximum of the residual variance. The PCA score plots can be used to observe the data distribution and structure of the data.

#### 2.4.2. DA

Discriminant Analysis (DA) is a supervised pattern recognition method where the number of categories and the samples that belong to each category are previously defined. In this study, DA was explored to classify *G. elata* from different origins based on predefined classes. Discrimination of the groups according the calculated Mahalanobis distance of a sample from their centers of gravity of the considered groups, so the greater the Mahalanobis distance between two given groups, the greater the spectral differences between them. Since the first 10 PCs cover the most variation contained in the spectral data, the DA was performed on the first 10 PCs resulting from the PCA applied on NIR spectral data recorded on *G. elata* samples.

#### 2.4.3. Si-PLS Model for Predicting PCC

The Si-PLS method proposed by Norgaard is a variable selection algorithm [22,23], which is an all-possible-subinterval-combination procedure test that contained relevant information related to the target parameter. The basic principle of Si-PLS is as follows: first, the full-spectrum region was spilt into different numbers of equal subintervals; second, the PLS regression model was constructed with different numbers and spectral subintervals; and lastly the combination of subinterval spectrums which has the lowest root mean square error of the cross validation (RMSECV) showed better performance [24].

In this study, the full spectrum (4000–10,000 cm^−1^) of *G. elata* samples was divided into 11, 12…, 25 intervals combined with two, three or four subintervals. The optimal combination of intervals and the number of PCs were optimized by leave-one-out cross validation and determined according to the lowest RMSECV. The final Si-PLS model’s performance was evaluated by the calibration set samples and tested by the prediction set samples.

Relationship between PCC and NIRS was also explored using a Si-PLS model. To establish a robust and high-performance regression model, it is important to ensure that the samples represent all types of variability as much as possible. The parameters as the root mean square error of the cross validation (RMSECV), root mean square error of the prediction (RMSEP), the coefficients of determination in the calibration data set (R_C_^2^) and the prediction data set (R_P_^2^) were all taken into account.

### 2.5. Software

In this study, a nonparametric Kruskal-Wallis test followed by Dun’s multiple comparison test was performed to analyze the significant difference among samples for the HPLC quantitative of four phenolic compounds content from different regions. These results were conducted using Graphpad Prism 7.0 (GraphPad Software, San Diego, CA, USA), the significance levels were set as *p* < 0.05. The clustering analysis was calculated using SPSS 18.0 (SPSS Inc., Chicago, IL, USA).

To establish the pattern recognition and prediction models, the PCA was carried out using SIMCA-P software (Umetrics AB, Umeå, Sweden), while the DA and the Si-PLS were carried out using TQ analyst software (Thermo Scientific Co.)

## 3. Results and Discussion

### 3.1. Optimization of Sample Extraction and Chromatogram for HPLC Method

To acquire more active ingredients and get better fingerprints of the samples, variables included in the extraction condition such as the solvent, methods and time were optimized by the orthogonal experiment shown in Table 2. We compared water, methanol and 50% alcohol as different extraction solvents, the results showed that diluted 50% alcohol had higher extraction efficiency and better fingerprints than others. Then we compared reflux, ultrasonic extraction and soak at room temperature as different methods, the results showed that there was no significance between two methods. However, ultrasonic extraction is chosen due to its time saving and easy operation. We next tried the sample extraction by ultrasonic treatment in diluted alcohol at 15 min, 30 min and 40 min, the results showed that effective extraction was get between 30 and 40 min. Therefore, the optimal extraction conditions for *G. elata* are presented in Section 2.2.

**Figure 1:**
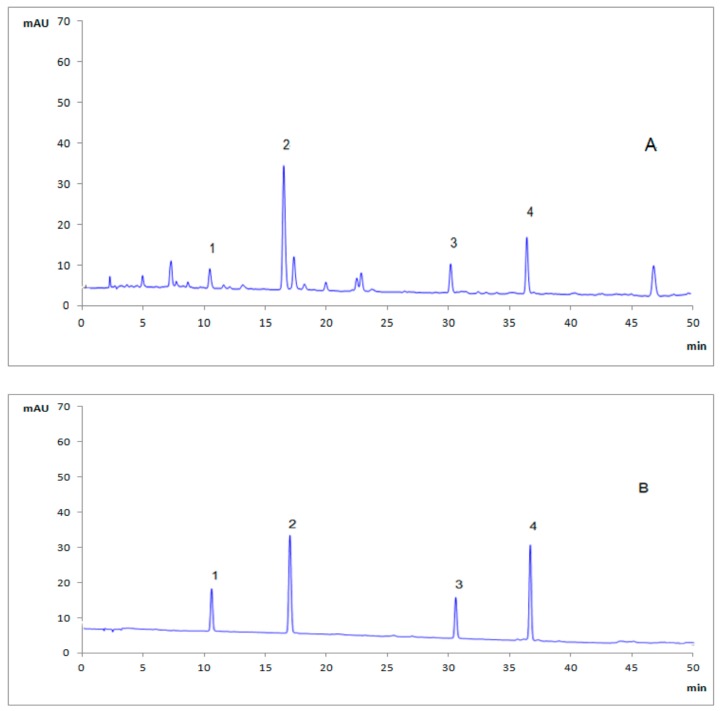
Typical HPLC chromatogram of sample solution of *G. elata* (**A**) and mixed standard solution (**B**). 1. GA, gastrodin; 2. HA, *p*-hydroxybenzyl alcohol; 3. PB, parishin B; 4. PA, parishin A.

In this study, four phenolic compounds (GA, HA, PB and PA) were marked as active ingredients to discriminate origins in *G. elata* samples. To ensure good separation performance within a short analysis time, the optimization of chromatographic conditions were also investigated and discussed as following: all sample analysis were carried on a Diamonsil C_18_ column (250 mm × 4.6 mm, 5 μm) at 30 °C, the mobile phase in this study consisted of 0.05% phosphoric acid solution (A) and acetonitrile (B) at a flow rate of 1.0 mL/min. A gradient program was set as: 0–10 min, 3–8%B; 10–18 min, 8–12%B; 18–40 min, 12–25%B; 40–50 min, 25–40%B. The detection wavelength was set at 220 nm, and the injection volume was defined to 10 μL. The typical HPLC chromatogram of *G. elata* sample was shown in Figure 1.

### 3.2. Quantitative Analysis of four PCC in G. elata Samples

**Figure 2:**
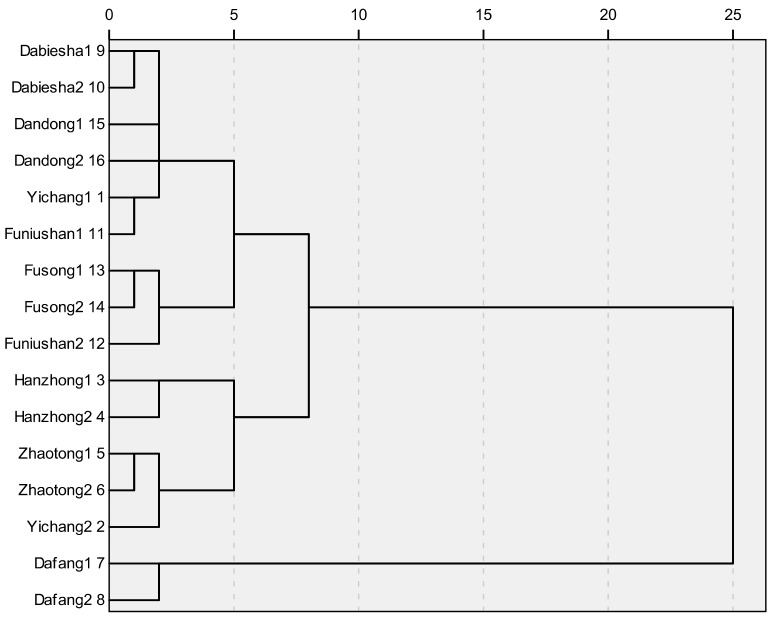
The clustering analysis of 16 batch *G. elata* samples.

The above-mentioned HPLC method was used to determine four PCC in *G. elata* samples from different origins simultaneously, and the results were shown in Table 3. It could be concluded that the contents of four analytes varied greatly with the sum of contents of GA and HA ranging from 3.37 mg/g to 7.43 mg/g, and the sum of contents of parishins (PA and PB) ranging from 3.89 mg/g to 9.32 mg/g. The results of statistical analysis showed that the four PCC was remarkably higher in samples from Dafang (Guizhou) compared to other origins. However, no clear separation was concluded among samples in terms of all different geographical origins. The clustering analysis of *G. elata* samples from 16 different origins was also calculated, which depended on the four phenolic compounds content in each sample using Euclidean distance in Figure 2. It could be concluded that the four main-cultivation areas could be discriminated separately mainly due to the difference came from chemical ingredients, while this marked-ingredients method was not able to reveal the chemical difference efficiently among samples from eight different cities, a newly-built NIRS method which was fast, non-destructive and more efficient was developed.

### 3.3. The NIR Spectra Analysis and Chemical Properties

**Figure 3:**
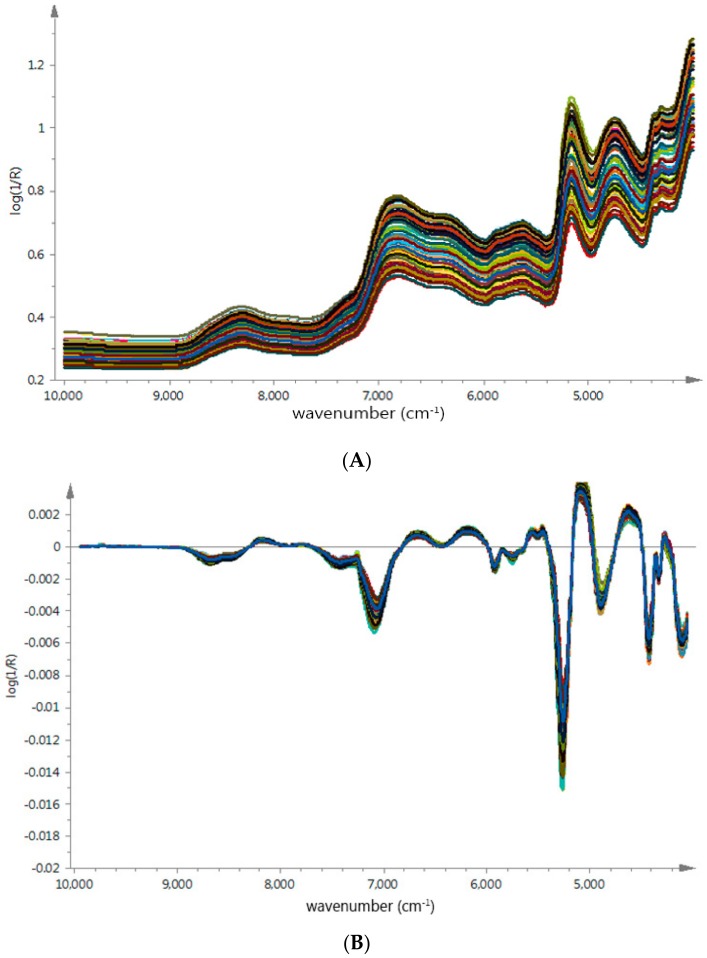
(**A**) The raw absorbance spectra of *G. elata* samples from different geographical origins; (**B**) Preprocessed spectra using Savitzky-Golay (SG) and FD pretreatment of *G. elata* samples from different geographical origins.

The average raw spectra of *G. elata* samples from different origins were shown in Figure 3A, which presented similar tendencies and indicated that samples contain similar chemical ingredients. We could observe strong absorptions at 4760 cm^−1^ to 5210 cm^−1^ and 4783 cm^−1^ to 4867 cm^−1^, related to the combination vibrations of O–H and a stretch and deformation of C–O group [25,26], respectively; these bond vibrations were probably corrected with PCC in *G. elata* samples [27,28]. However, due to physical properties such as particle size effect and numerous chemical ingredients in herb medicines, it was difficult to find specific region based on geographical origin.

In order to remove baseline drift and enhance the absorbance of feature spectral, the SG algorithm and FD were used to make the unique spectra features stand out. As shown in Figure 3B, the sample spectrum difference were observed in several regions, around 4900 cm^−1^ and 8500 cm^−1^. Especially the spectra range between 4600 cm^−1^ and 5200 cm^−1^ related to the interaction between O–H and aromatic ring [29,30], these spectrum regions could probably be applied to build identification models more efficiently.

Moreover, further spectral transformations needed to be tried, such as MSC and SNV, to remove slope variation and correct scatter effect, FD/SD and SG to enhance the main absorbance features in spectra. These feature spectra regions will be vital for classification, the detail information was explored in Section 3.5 in this study.

### 3.4. Data Processing and Splitting

With different spectra preprocessed, the data were partitioned into a calibration set and validation set, respectively. The former was used to establish the calibration model, the latter was used to test the prediction ability of the model constructed. The Kennard and Stone (K-S) algorithm which can ensure that both sets were well proportioned was adopted and its principle is to cover the space in a uniform manner by maximizing the Euclidean distances between selected and remaining objects. Finally, 10 batches from 16 batches (100 samples) were selected as calibration set, and the remaining (60 samples) were taken as validation set. The mean, range and standard deviation for the parameters measured in both sets are listed in Table 4, which indicated that these samples were all appropriately distributed.

### 3.5. Development of Discrimination Models

To compare the performance of different classification models in this study, two pattern recognition method PCA and DA were established using the selected calibration and validation set. PCA was established to extract effective data and main information to examine qualitative difference between two kinds of samples, all the samples’ spectra were used for the PCA model; while DA can be performed to establish a classification model that related to the characteristic sample spectral data by Mahalanobis-distance calculation.

Different transformation methods for spectra such as MSC, SNV, SG and FD/SD were compared and the best data transformation was found in this study.

#### 3.5.1. Optimization of NIRS Transfomation Conditions for PCA Model

The spectra data should be transformed for an optimal performance before the calibration stage. The raw and all transformed spectra methods were compared in this study, which revealed that the MSC and FD was the optimal condition for further analysis. MSC was used to remove physical spectral effect as particle size and noise, FD was used to reduce data dimensions so that PCA could be established based on mainly chemical spectral information. 

#### 3.5.2. The Establishment of PCA Model

**Figure 4:**
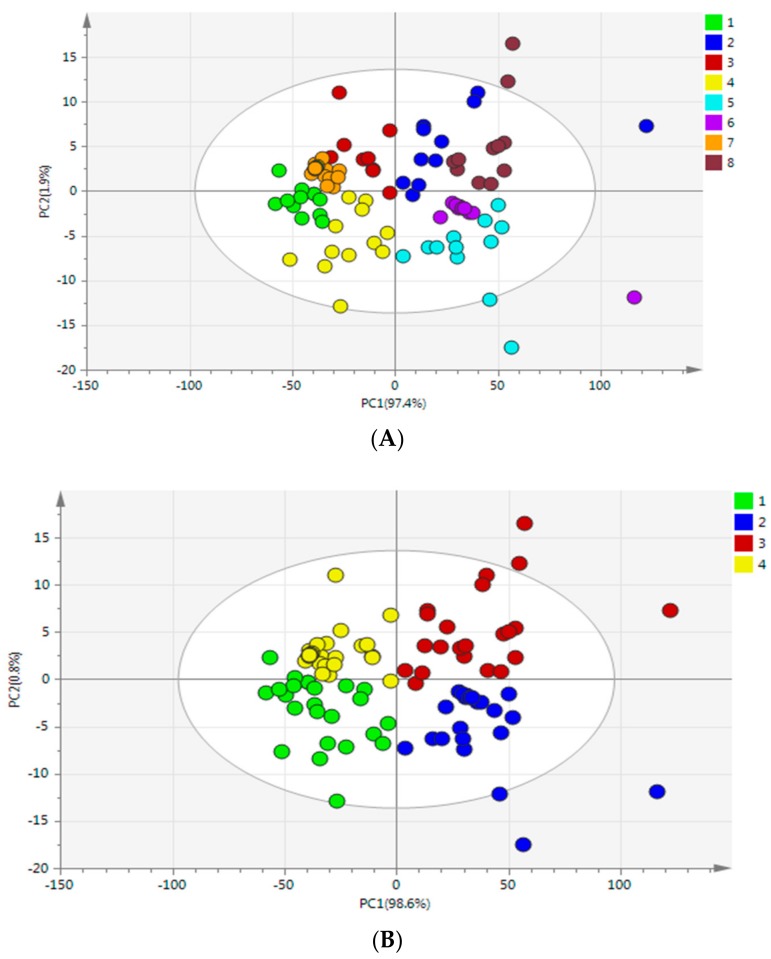

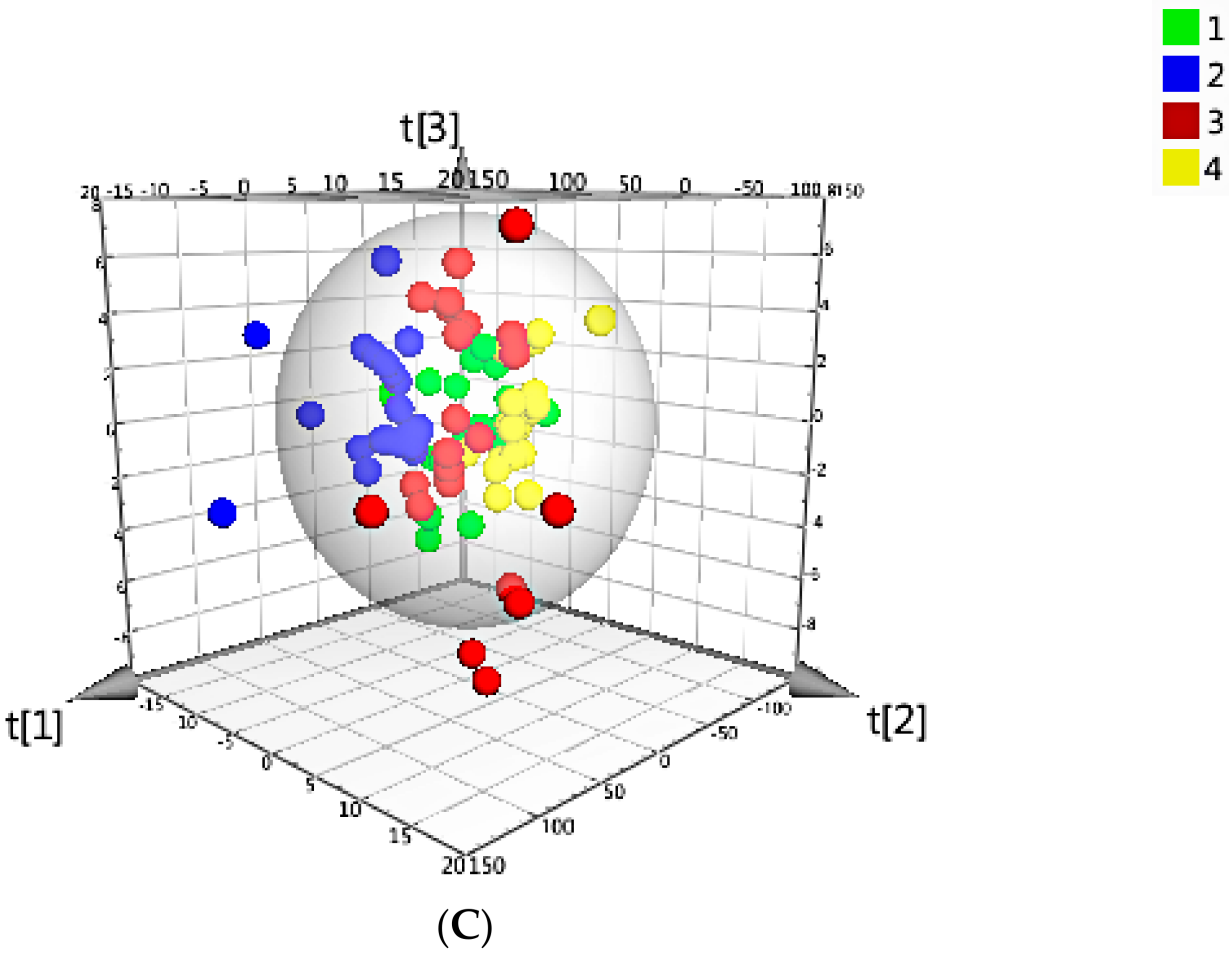
(**A**) Principal component score plot of PC1 and PC2 based on near infrared (NIR) spectra of *G. elata* samples from eight different cities: 1. Yichang (Hubei) 2. Dabieshan (Anhui) 3. Zhaotong (Yunnan) 4. Hanzhong (Shanxi) 5. Fusong (Jilin) 6. Dandong (Liaoning) 7. Dafang (Guizhou) 8. Funiushan (Henan); (**B**) Principal component score plot of PC1 and PC2 based on NIR spectra of *G. elata* samples from four main-cultivated areas: 1. Yangtse Gorges and Shennongjia area; 2. Northeast provinces area; 3. Dabieshan area; 4. Southwest provinces area; (**C**) Three-dimensional score plot using PC1, PC2, and PC3 for discriminating *G. elata* from four main-cultivated areas: 1. Yangtse Gorges and shennongjia area; 2. Northeast provinces area; 3. Dabieshan area; 4. Southwest provinces area.

PCA was used to decrease the data to a small number of PCs, the PCs represent most of the original data that were used to extract useful information from raw spectra data. Figure 4A showed the score plot of the first two PCs which account for 99.3% of the variation in the NIR spectra of *G. elata* samples from eight different cities. However, the samples’ separation was not all clear and some overlap existed among the groups. As we can see, alone the PC1 (97.4%), the samples were totally separated from different origins, and four areas were also clustered separately, while the samples from Dafang city and Zhaotong city were partly separated along the PC2 (1.9%). It could be concluded that: (1) there were inherent compositional differences among samples from eight different cities though they came from same species; and (2) samples were similar from the same province in terms of their chemical composition, since the environmental conditions such as topography, soil and moisture of different cities in same province are similar. Therefore, further chemometric methods were needed to classify different *G. elata* samples.

However, Figure 4B clearly showed a score plot of *G. elata* samples from four different main-cultivate areas represented by PC1 (98.6%) and PC2 (0.8%), the different categories from four main-cultivate areas separated individually indicated that they have unique features probably due to the different cultivated areas with specific growing conditions such as soil and altitude; while the three-dimensional score plot using PC1, PC2, and PC3 for discriminating *G. elata* from four main-cultivated areas was shown in Figure 4C, which performed barely satisfactory than PC1 vs. PC2.

**Figure 5:**
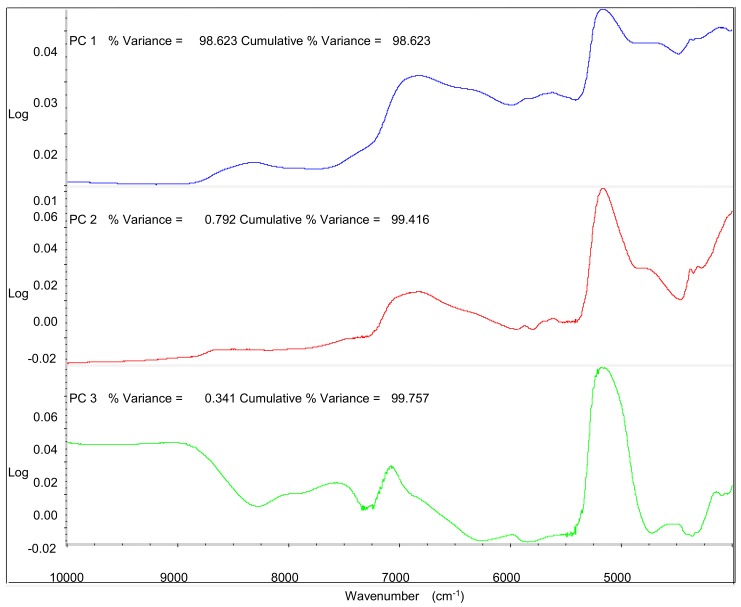
The correlative near infrared spectrum (NIRS) regions used for the classification of four different main-cultivated areas of PC1, PC2 and PC3.

In order to account for the specific regions that used to distinguish the samples from different cultivation areas, the first three PCs that explained 99.8% of the total variance were investigated to discuss in Figure 5. PC1 had the highest correlation between 4800 cm^−1^ and 7200 cm^−1^ in spectrum, which related to O–H overtones [31,32,33]; PC2 had the highest correlation between 4200 cm^−1^ and 5200 cm^−1^ in spectrum, which related to stretch and deformation of C–O group [34,35,36]; PC3 had the highest correlation between 4800 cm^−1^ and 5200 cm^−1^ in spectrum, which related to interaction between O–H and aromatic ring [37,38]; These results could be concluded that the differences of chemical components including the phenolics and water might account for the classification of their geographical origin. 

#### 3.5.3. Optimization of NIRS Transformation Conditions for DA Model

Different data transformation was also used for an optimal performance to build DA model. The raw and all transformed spectra were compared in this study in Table 5, which could be concluded that the MSC and SD was the optimal condition for further analysis, the models discriminate *G. elata* samples with the accuracy range from 60% to 98%.

#### 3.5.4. The Establishment of DA Model

**Figure 6:**
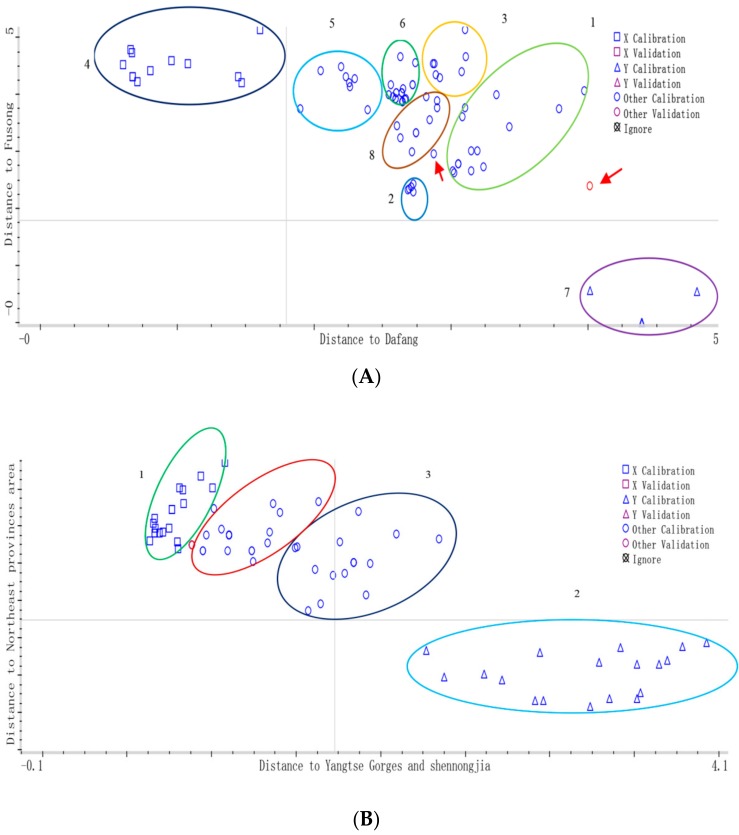
(**A**) DA scores plot of *G. elata* samples marked by different geographical origins from eight differentcities: 1. Yichang (Hubei) 2. Hanzhong (Shanxi) 3. Zhaotong (Yunnan) 4. Dafang (Guizhou) 5. Dabieshan (Anhui) 6. Funiushan (Henan) 7 Fusong (Jilin) 8. Dandong (Liaoning). The red arrows referred to sample that misclassified in calibration sets; (**B**) DA scores plot of *G. elata* samples marked by different geographical origins. From four main-cultivated areas: 1. Yangtse Gorges and Shennongjia area; 2. Northeast provinces area; 3. Dabieshan area; 4. Southwest provinces area.

DA model was performed on the first 10 PCs of all samples to classify *G. elata* samples from eight different cities in Figure 6A, which accounted for 99.9% variation of the whole spectrum and the total correct classification rate was 98%.

For the calibration set, there was one sample of main-cultivated origin from the “Yangtse Gorges and Shennongjia area”, of which Yichang (Hubei) was identified as “Dandong (Liaoning)”; next is the Northeast area group, where one sample also was wrongly classified; for the other varieties, all the samples were classified correctly. The percentage of correctly identified varieties was 98% for the calibration set. These misclassifications probably due to chemical components varied slightly in samples from different origins; while for validation set, only one sample of “Zhaotong (Yunnan)” was misclassified, others were correctly identified, the percentage of correctly identified varieties was 99% for the validation set.

In a similar way, DA model was performed on the first 9 PCs of all samples to classify *G. elata* samples from four different main-cultivate areas, which accounted for 99.9% variation of the whole spectrum and the total correct classification rate was 98% in Figure 6B. The percentage of correctly identified varieties was 97% and 99% for the calibration and validation set, respectively. In order to compare two established discrimination models PCA and DA for the 16 batches *G. elata* samples in this study, the discrimination results (rate%) were shown in Table 6.

Overall, the geographical origins of *G. elata* samples could be effectively discriminated by using NIRS combined with the DA method, which may be sufficiently sensitive to be a practical utility. 

### 3.6. Development of PCC Prediction Model

Since the phenolic compounds content (PCC) contained in *G. elata* is an important parameter for quality evaluation, the correlation between the PCC and NIRS was also developed. In this study, PCC included four parameters (GA, HA, PA and PB) to be explored. The *G. elata* sample parameters (mean, range and standard deviation) of the calibration and validation sets are listed in Table 2 before. It was indicated that samples were distributed appropriately in the calibration and validation sets.

**Figure 7:**
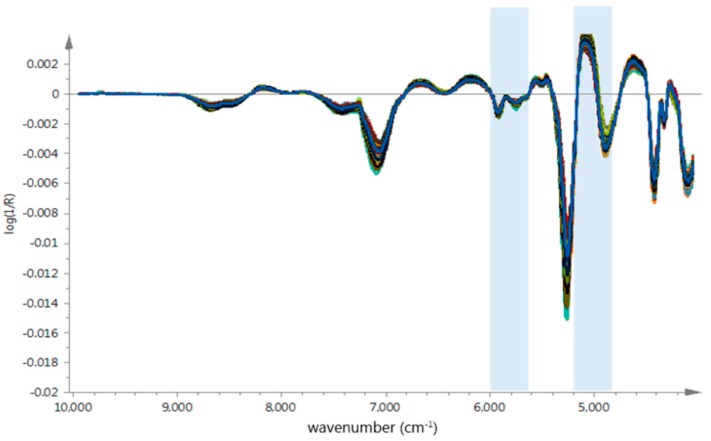
The optimal spectral sub-intervals selected for *G. elata* samples’ Si-PLS model establishment.

**Figure 8:**
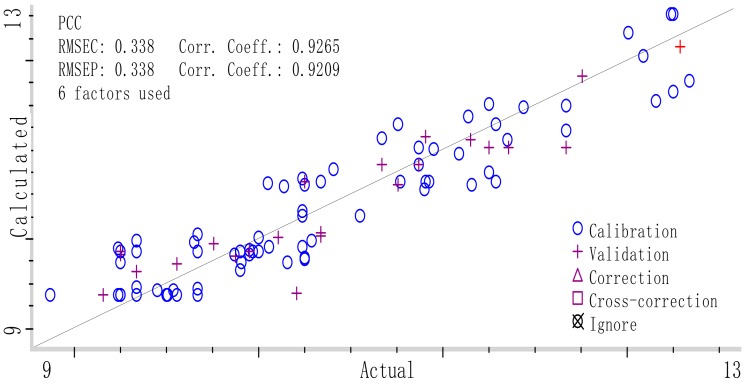
Reference measured versus NIR prediction by synergy interval partial least squares (Si-PLS) in calibration and prediction set of *G. elata* samples.

**Figure 9:**
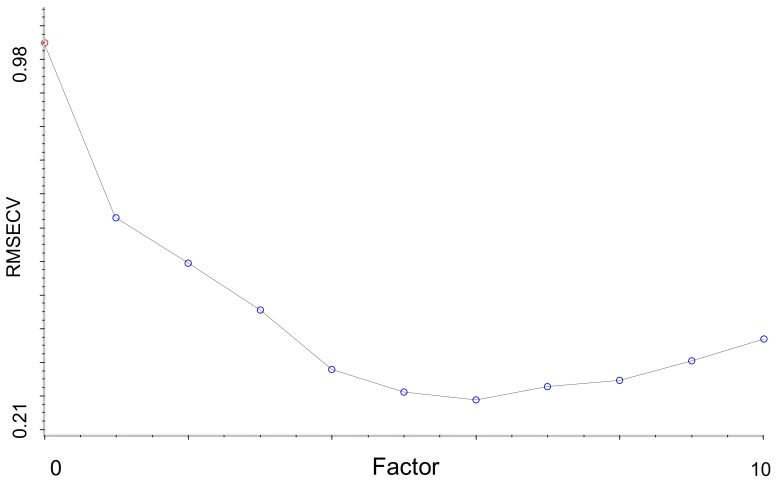
Plot of root mean square error of the cross validation (RMSECV) versus number of factors selected by Si-PLS for PCC.

The best PLS calibration model for PCC prediction was obtained with MSC and SD spectrum pre-treatment in the full wavelength range. As we described in Section 2.4.3, Si-PLS is an efficient variable selection methods which has shown the potential for modeling with good precision used in some research [39,40,41], in this study, the optimal Si-PLS model for PCC prediction from different origins was obtained with the combination of 2 subintervals when the spectrum was divided into 15 subintervals as shown in Table 7, and the optimal chosen combination subintervals correspond to 4800 cm^−1^ to 5200 cm^−1^ and 5600 cm^−1^ to 6000 cm^−1^ in Figure 7. The parameters for optimal Si-PLS model was as follows: R_C_^2^ was 0.9265 and RMSECV 0.338 in calibration set, the R_P_^2^ was 0.9209 and RMSEP 0.338 in validation set. The established optimal Si-PLS model was shown in Figure 8 and the plot of RMSECV versus number of factors selected by Si-PLS for PCC was shown in Figure 9.

It has been known that NIR is based on molecular vibration and often overlapped to find feature spectra origins. One of the selected intervals by Si-PLS 4800 cm^−1^ to 5200 cm^−1^ was relate to the aromatic ring absorptions of in basic phenolic compound structure [29,30,31,32] and thus indicated that NIR could be a potential quality-evaluation tool for PCC prediction in *G. elata* samples.

### 3.7. Comparison between HPLC and NIR

To evaluate the TCM quality, numerous methods, such as HPLC, have been applied to establish and prove the quality of medicine. Both HPLC and NIR methods are important due to their individual characteristics. However, compared to target-marker HPLC analysis of *G. elata* samples from different origins, our study showed that the NIR method developed was more effective and reliable for the classification of *G. elata* samples. The fast, low cost and non-destructive NIR method coupled with pattern recognition and quantitative PCC evaluation could be helpful in the discrimination of *G. elata* from different geographic origins. Nevertheless, it was just a first step and more representative samples should be collected to make the models robust.

From the above results, it could be concluded that the NIR method developed, combined with multivariate analysis, is practical for the quality evaluation of *G. elata* and may also be applicable to other herbal medicines similarly.

## 4. Conclusions

This study provided deep insights into the quality evaluation profile of *G. elata* samples from different geographical origins based on the target-marker HPLC method and non-destructive NIR method. It was validated that NIR, coupled with pattern recognition and quantitative methods, could be a potential tool to control the quality of *G. elata* in an accurate and efficient way. This study showed that differences among *G. elata* samples from various origins do exist and a high-performance classification can be obtained by optimal DA method with the accuracy up to 99%, NIR spectroscopy combined with optimal Si-PLS model can provide an accurate prediction of PCC in *G. elata*. These results are a preliminarily demonstration of the potential use of NIRS combined with chemometrics as a rapid and effective method for discriminating the geographical origin and predicting the PCC of *G. elata.* Moreover, this finding will also provide useful information for the standardized plantation of *G. elata*. However, more samples will be needed to confirm these results and develop more robust models for prediction in further studies.

## Figures and Tables

**Table 1:** The sampling regions of *G. eleta.*

Batch No.	Geographic Origin	Sample No.	Site	Harvesting Time
1	Yangtse Gorges and shennongjia area	1–10	Yichang (Hubei)	2017.12
2		11–20		2018.1
3		21–30	Hanzhong (Shanxi)	2017.12
4		31–40		2018.1
5	Southwest provinces area	41–50	Zhaotong (Yunnan)	2017.12
6		51–60		2018.1
7		61–70	Dafang (Guizhou)	2017.12
8		71–80		2018.1
9	Dabieshan area	81–90	Dabieshan (Anhui)	2017.12
10		91–100		2018.1
11		101–110	Funiushan (Henan)	2017.12
12		111–120		2018.1
13	Northeast provinces area	121–130	Fusong (Jilin)	2017.12
14		131–140		2018.1
15		141–150	Dandong (Liaoning)	2017.12
16		151–160		2018.1

**Table 2:** Orthogonal experiment table of *G. elata*’s extraction technology.

	A (Solvent)	B (Method)	C (Time)	D (Column)
1	water	ultrasonic	30 min	C_18_
2	water	reflux	40 min	C_18_
3	water	soak at room temperature	15 min	C_18_
4	methanol	soak at room temperature	40 min	C_18_
5	methanol	ultrasonic	15 min	C_18_
6	methanol	reflux	30 min	C_18_
7	diluted	reflux	15 min	C_8_
8	diluted	soak at room temperature	30 min	C_8_
9	diluted	ultrasonic	40 min	C_8_

**Table 3:** Contents of four phenolic compounds in 16 batches of *G. elata* samples (*n* = 10).

Batch No.	Site	GA (mg/g)	HA (mg/g)	PB (mg/g)	PA (mg/g)	Total (mg/g)
1	Yichang (Hubei)	0.35 ± 0.01	3.29 ± 0.11	1.09 ± 0.01	5.19 ± 0.01	9.92 ± 0.02
2		0.40 ± 0.03	4.01 ± 0.12	1.21 ± 0.02	6.01 ± 0.03	11.63 ± 0.21
3	Hanzhong (Shanxi)	0.52 ± 0.03	3.11 ± 0.14	0.92 ± 0.03	6.99 ± 0.16	11.54 ± 0.11
4		0.61 ± 0.04	4.39 ± 0.10	0.96 ± 0.04	6.62 ± 0.17	12.29 ± 0.12
5	Zhaotong (Yunnan)	0.59 ± 0.02	5.19 ± 0.11	1.30 ± 0.07	5.20 ± 0.10	12.31 ± 0.19 *
6		0.51 ± 0.01	5.02 ± 0.10	1.29 ± 0.01	6.33 ± 0.13	13.24 ± 0.21 *
7	Dafang (Guizhou)	1.20 ± 0.01	6.11 ± 0.09	1.49 ± 0.14	7.01 ± 0.12	15.90 ± 0.31 *
8		1.09 ± 0.01	6.34 ± 0.05	1.32 ± 0.15	7.99 ± 0.11	16.51 ± 0.22 *
9	Dabieshan (Anhui)	0.45 ± 0.02	4.09 ± 0.12	1.06 ± 0.01	4.29 ± 0.12	9.90 ± 0.13
10		0.50 ± 0.07	4.21 ± 0.11	1.09 ± 0.03	4.03 ± 0.03	9.87 ± 0.14
11	Funiushan (Henan)	0.28 ± 0.01	3.09 ± 0.01	0.99 ± 0.01	5.09 ± 0.04	9.51 ± 0.15
12		0.39 ± 0.02	3.21 ± 0.03	0.69 ± 0.01	4.69 ± 0.01	8.79 ± 0.21
13	Fusong (Jilin)	0.39 ± 0.01	3.29 ± 0.04	0.69 ± 0.05	3.09 ± 0.06	7.32 ± 0.11 ^▲^
14		0.29 ± 0.02	4.01 ± 0.02	0.59 ± 0.06	3.29 ± 0.17	8.23 ± 0.14 ^▲^
15	Dandong (Liaoning)	0.39 ± 0.01	3.29 ± 0.11	1.21 ± 0.04	4.09 ± 0.10	8.79 ± 0.21 ^▲^
16		0.29 ± 0.01	3.29 ± 0.09	0.99 ± 0.06	3.29 ± 0.09	7.68 ± 0.10 ^▲^

* Compared to other three main-cultivated regions, there was a prominent significance in these groups (*p* < 0.05). ^▲^ Compared to Southwest provinces area main-cultivated regions, there was a prominent significance in these groups (*p* < 0.05). The data showed in table were expressed as Mean ± SD.

**Table 4:** Division and parameters of samples in the calibration and validation set (mg/g).

Parameters	Subsets	S.N. ^a^	Range	Mean	S.D. ^b^
GA	calibration set	100	0.26–1.28	0.72	2.1
	validation set	60	0.22–1.24	0.76	1.9
HA	calibration set	100	3.02–6.54	4.75	2.5
	validation set	60	3.01–6.49	4.65	2.2
PB	calibration set	100	0.63–1.29	0.92	1.6
	validation set	60	0.60–1.27	0.91	1.8
PA	calibration set	100	3.02–8.21	5.62	2.0
	validation set	60	3.01–8.32	5.82	2.5
PCC (total)	calibration set	100	7.03–16.98	12.91	4.6
	validation set	60	7.21–16.56	12.43	4.2

^a^ S.N., the number of samples; ^b^ S.D., standard deviation.

**Table 5:** Numbers that had been incorrectly classified for *G. elata* samples using discriminant analysis (DA) models on the raw spectra and those with various transformation (*n* = 100).

Data Pretreatment	Eight Different Cities		Four Main-Cultivate Origins	
	Calibration Set	Correct Percent (%)	Calibration Set	Correct Percent (%)
RAW	35 (5) ^a^	60	28 (7)	65
MSC	23 (2)	75	22 (8)	70
SNV	20	80	15	85
MSC + FD	15	85	10 (9)	81
SNV + FD	13	87	8	92
MSC + SD	2	98	3	97
SNV + SD	5	95	10	90

^a^ The number in brackets represents the number of outlier samples.

**Table 6:** Discrimination results (rate%) of established principal component analysis (PCA) and DA models for the *G. elata* validation set.

Geographic Origin	Validation Set (%)		Cities	Validation Set (%)	
	PCA	DA		PCA	DA
Yangtse Gorges and shennongjia area	90	98	Yichang (Hubei)	90	95
			Hanzhong (Shanxi)	93	98
Southwest provinces area	91	95	Zhaotong (Yunnan)	91	97
			Dafang (Guizhou)	90	98
Dabieshan area	93	98	Dabieshan (Anhui)	91	97
			Funiushan (Henan)	92	99
Northeast area	90	99	Fusong (Jilin)	93	98
			Dandong (Liaoning)	92	99

**Table 7:** Results of Si-PLS models with selected optimal spectral subintervals for PCC.

Number of Subintervals	Selected Subintervals	PCs	R_C_^2^	RMSECV	R_P_^2^	RMSEP
11	[1 3]	8	0.8995	0.534	0.8923	0.657
12	[3 7]	7	0.8997	0.589	0.8976	0.602
13	[4 5]	8	0.9021	0.498	0.9128	0.592
14	[3 8]	6	0.9011	0.482	0.9010	0.438
15	[2 4]	6	0.9265	0.338	0.9209	0.338
16	[3 6 8]	9	0.9162	0.432	0.9121	0.429
17	[4 7 9]	8	0.9076	0.412	0.9056	0.403
18	[3 9 11]	7	0.9129	0.398	0.9117	0.376
19	[2 5 12]	7	0.9245	0.388	0.9232	0.365
20	[4 9 15]	8	0.9211	0.401	0.9121	0.398
21	[6 8 14]	9	0.9234	0.385	0.9136	0.374
22	[3 8 13]	8	0.9019	0.376	0.9002	0.365
23	[7 9 14]	8	0.8992	0.402	0.8945	0.398
24	[4 9 15]	6	0.9128	0.343	0.9109	0.312
25	[5 8 11 14]	6	0.9211	0.351	0.9145	0.326
